# Spontaneous Graft‐Induced Dyskinesias Are Independent of 5‐HT Neurons and Levodopa Priming in a Model of Parkinson's Disease

**DOI:** 10.1002/mds.28856

**Published:** 2021-11-12

**Authors:** Emma L. Lane, David J. Harrison, Elena Ramos‐Varas, Rachel Hills, Sophie Turner, Mariah J. Lelos

**Affiliations:** ^1^ School of Pharmacy and Pharmaceutical Sciences Cardiff University Cardiff United Kingdom; ^2^ Brain Repair Group, School of Biosciences Cardiff University Cardiff United Kingdom

**Keywords:** Parkinson's disease, dopamine, cell therapy, graft‐induced dyskinesias, neuroinflammation, microglia, 5‐HTl‐dopa

## Abstract

**Background:**

The risk of graft‐induced dyskinesias (GIDs) presents a major challenge in progressing cell transplantation as a therapy for Parkinson's disease. Current theories implicate the presence of grafted serotonin neurons, hotspots of dopamine release, neuroinflammation and established levodopa‐induced dyskinesia.

**Objective:**

To elucidate the mechanisms of GIDs.

**Methods:**

Neonatally desensitized, dopamine denervated rats received intrastriatal grafts of human embryonic stem cells (hESCs) differentiated into either ventral midbrain dopaminergic progenitor (vmDA) (n = 15) or ventral forebrain cells (n = 14).

**Results:**

Of the eight rats with surviving grafts, two vmDA rats developed chronic spontaneous GIDs, which were observed at 30 weeks post‐transplantation. GIDs were inhibited by D_2_‐like receptor antagonists and not affected by 5‐HT1A/1B/5‐HT6 agonists/antagonists. Grafts in GID rats showed more microglial activation and lacked serotonin neurons.

**Conclusions:**

These findings argue against current thinking that rats do not develop spontaneous GID and that serotonin neurons are causative, rather indicating that GID can be induced in rats by hESC‐derived dopamine grafts and, critically, can occur independently of both previous levodopa exposure and grafted serotonin neurons. © 2021 The Authors. *Movement Disorders* published by Wiley Periodicals LLC on behalf of International Parkinson and Movement Disorder Society

Clinical trials using fetal dopamine (DA) cells as a neuroreparative strategy for Parkinson's disease (PD) have produced remarkable long‐term recovery of function in some patients^1^ and graft survival for over 20 years.^2^ However, the results have been variable and a proportion of patients in three major trials developed persistent abnormal involuntary movements (AIMS), now termed “graft‐induced dyskinesias” (GIDs), occurring in the absence of levodopa (l‐dopa).^3^, ^4^, ^5^ Pre‐existing l‐dopa induced dyskinesias (LID) have been identified as a potential risk factor for the development of GIDs, but other potential risk factors include aberrant immune responses,^6^, ^7^ incomplete graft innervation,^8^ 5‐HT neurons in the graft,^9^ abnormal ratios of 5‐HT/DA receptors^10^, ^11^, ^12^ or activity of specific 5‐HT receptors.^13^ Investigations of the mechanisms underlying GIDs have been hampered by rodent models that do not replicate the spontaneous nature of the behaviors. Rather they rely on administration of l‐dopa or amphetamine to generate acute, transient GID expression^14^, ^15^ in transplanted animals.

With the evolution of human stem cell (hESC)‐derived sources of cells for transplantation, it is now possible to generate ventral midbrain DA (vmDA) grafts free of 5‐HTergic neurons and to assess if GIDs can occur also in the absence of 5‐HT. Intrastriatal grafts of hESC‐derived vmDA cells have been widely reported to survive, integrate, release DA, and alleviate functional impairments in rodent models of PD^16^, ^17^, ^18^, ^19^ but GIDs have not been explored. To ensure the safety of new cell products for transplantation, it is imperative that we evaluate and understand the potential risk of side effects.

In the process of studying the long‐term functional efficacy of hESC‐derived vmDA grafts in 6‐hydroxydopamine (6‐OHDA) lesioned rats, we unexpectedly observed spontaneous, continuous abnormal AIMs in a subset of rats. The rats had been checked weekly for health and welfare purposes, but detailed behavioral analysis commencing at 30 weeks led to the identification of chronic GIDs. We characterized the GIDs pharmacologically, targeting DAergic and 5‐HTergic systems. Subsequent immunohistochemical analysis of the brain tissue compared vmDA grafts from GID rats with vmDA grafts from non‐GID rats and with control hESC‐derived non‐DA, forebrain‐patterned (vFB) cells.^17^

## Materials and Methods

Experiments were conducted in compliance with the United Kingdom (UK) Animals (Scientific Procedures) Act 1986 under Home Office License No. 30/2498, with the approval of the local Cardiff University Ethics Review Committee.

**FIG. 1:**
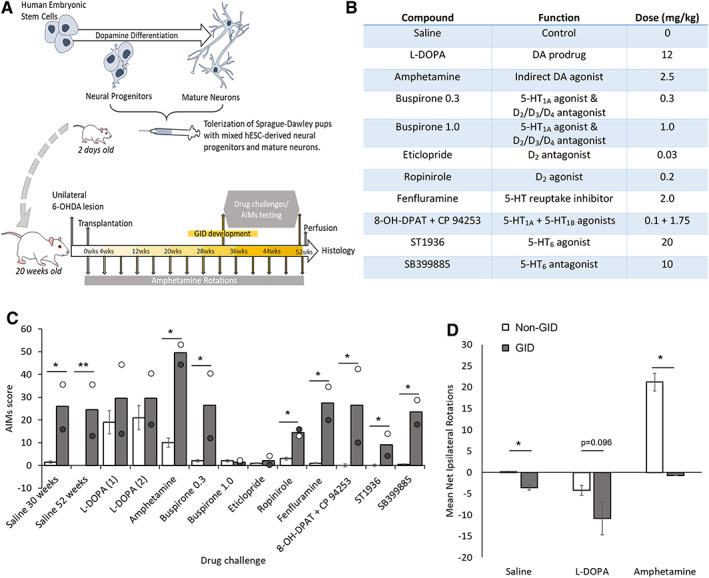
(**A**) Schematic representation of the timeline of experimental events. (**B**) Table of pharmacological agents used during abnormal involuntary movement (AIMs) analysis in rats. (**C**) Mean and individual AIMs score for vmDA graft‐induced dyskinesias (GID) rats (n = 2). Mean AIMs score for non‐GID rats includes small vmDA grafts and vFB grafts, combined into “non‐GID” dataset (n = 6), in response to saline or drug challenges. AIMs were stable between 30 and 52 weeks post‐graft (both saline datasets, *P* < 0.05 for non‐GID vs. GID rats). Non‐GID rats demonstrate AIMs in response to l‐dopa, but no change in AIMs is evident in GID rats. For GID rats, 0.3 mg/kg buspirone (5‐HT1A agonist/D2‐like antagonist), fenfluramine (5‐HT reuptake inhibitor), 8‐OH‐DPAT+CP94253 (5‐HT1A/1B agonists), and SB399885 (5‐HT6 antagonist) did not alter expression of the AIMs behaviors (all ps < 0.05 for non‐GID vs. GID). High dose buspirone and eticlopride both abolish AIMs (ps = n.s. for non‐GID vs. GID), without affecting normal locomotion. (**D**) Mean net rotations to the ipsilateral (positive scale) and contralateral (negative scale) sides for non‐GID and GID rats in response to saline (*P* < 0.05 for non‐GID vs. GID rats), l‐dopa (*P* = 0.096) or amphetamine (*P* < 0.05). In contrast to non‐GID rats, GID rats did not rotate in response to amphetamine, but did rotate spontaneously and in response to l‐dopa. Note: All rats had complete unilateral lesions, as evidenced by their pre‐graft rotational response to amphetamine (non‐GID rats: mean = 12.3 net turns/minute, SEM = 0.95; GID rats: mean = 14.5 net turns/minute, SEM = 1.27) and the loss of nigral TH^+^ neurons (non‐GID rats: mean = 99.5%; loss, SEM = 0.17; GID rats: mean = 99.2% loss, SEM = 0.18). vFB = ventral forebrain control grafts; vmDA = ventral midbrain ventral mesencephalic grafts. Error bars = standard errors of the mean. *P ≤ 0.05, **P ≤ 0.01.

Sprague–Dawley rat pups (female, n = 29) were neonatally desensitized at 2 days post‐birth with mixed hESC‐derived neural progenitors and mature neurons (Fig. 1A), as described elsewhere.^20^ Rats were housed in groups of 3–4, with a 14‐hour:10‐hour light:dark cycle. At 20 weeks old, rats received MFB 6‐OHDA lesions, as previously described.^18^ Rats were sorted into matched groups based on amphetamine‐induced rotations (2.5 mg/kg) conducted at 4 weeks post‐lesion (mean net rotations/minutes: vmDA = 15.3 ± 0.8; vFB = 14.6 ± 1.0). Rats received intrastriatal transplants of hESC‐derived cells (vmDA or vFB).^17^ H9 cells were differentiated for 16 days and made into a suspension of 60,000 cells/μL, as previously described.^17^ For both transplanted groups, 4 μL (240,000 total cells/graft) was injected into the neostriatum at the following coordinates: (1) AP: +0.5, ML: −3.0; (2) AP: +1.2, ML: −2.7; (3) DVs: −4/−5.

Rotational behaviors were measured for 90 minutes (Rotorat, Med Associates) after 2.5 mg/kg methamphetamine (Sigma, cat. no. M8750), for 60 minutes after 0.05 mg/kg apomorphine (Sigma, cat. no. Y0001465) and after administration of other pharmacological agents (Fig. 1B). Observations from 30 weeks post‐graft revealed spontaneous AIMs. AIMs (as described in Breger et al)^21^ were scored every 10 minutes for 60 minutes post‐administration of saline or drug. The assessor was blind to both the substance administered and the rat group allocation. During AIMs scoring, the experimenter noted whether normal locomotion and exploratory behaviors were evident in the rotameters, to give confidence that any reduction in GID expression was not the consequence of reduced activity overall. Rotational data were collected in an unbiased manner using automated rotometers.

At 52 weeks post‐graft, rats were terminally anaesthetized and transcardially perfused (4% paraformaldehyde [PFA]). Brains were processed for peroxidase‐based immunohistochemistry as previously described.^18^ CD4^+^, CD8^+^, and 5‐HT^+^ cells were counted manually within and at the border of the graft. TH^+^, HuNu^+^, Ox42^+^/CD11b^+^, and GFAP^+^ cells were estimated using unbiased stereology. Two‐dimensional stereology was performed (Olympus BX50 microscope, Olympus C.A.S.T. image‐analysis software). Ox42 was also measured by optical density (ImageJ, NIH).

Pharmacological challenges and rotation data were analyzed using Kruskal‐Wallis non‐parametric test with Group (non‐GID vs. GID) as the factor. Histological data were analyzed by one‐way ANOVA with Group (vFB, vmDA non‐GID, and vmDA GID) as the factor. Histological and behavioral data were correlated using Spearman's ρ. All statistical analyses were conducted using IBM SPSS Statistics 25 software.

## Results

### Dopaminergic Grafts Can Induce Spontaneous GIDs

To obtain long‐term survival of human grafts in a rodent model without the need for daily immunosuppression, we applied a model of neonatal desensitization in rats, which required subcutaneous injection of human cells at P0‐5.^20^, ^22^ In adulthood, the desensitized rats received unilateral lesions and subsequently hESC‐derived vmDA or vFB intrastriatal transplants. Only a subset of the transplanted rats (4/15) had surviving vmDA grafts and, of these, two displayed spontaneous contralateral rotations, a trend for l‐dopa‐induced rotations and reduced amphetamine‐induced rotations (Fig. 1D). These two rats also developed spontaneous AIMs at 30 weeks post‐graft (Fig. 1C and Supporting Data). These were compared to rats with smaller surviving vmDA grafts (n = 2) and rats with non‐DA, vFB grafts (n = 4/14) (neither the smaller vmDA nor the vFB graft groups displayed spontaneous AIMs or reduced amphetamine‐induced rotations).

To elucidate the neurobiological basis of the GIDs, pharmacological challenges were undertaken using receptor agonists and antagonists. AIMs were observed in both the home cage and rotometers (Supplementary Video S1), and scored blind twice post‐saline administration (at 30 and 52 weeks post‐graft) and twice post‐l‐dopa administration (Fig. 1C). These sequential observations suggest that GIDs were chronic, stable, and minimally affected by stress or repeated exposure to rotometers. For GID rats, 0.3 mg/kg buspirone (5‐HT_1A_ agonist/D_2_‐like antagonist), fenfluramine (5‐HT reuptake inhibitor), 8‐OH‐DPAT+CP94253 (5‐HT_1A/1B_ agonists) and SB399885 (5‐HT_6_ antagonist) did not alter expression of the AIMs behaviors. High dose 1 mg/kg buspirone^23^ and eticlopride (D_2_/D_3_ antagonist) largely eliminated GID expression without reducing normal motor activity.

### Larger vmDA Grafts Induce Neuroinflammation and GIDs

**FIG. 2:**
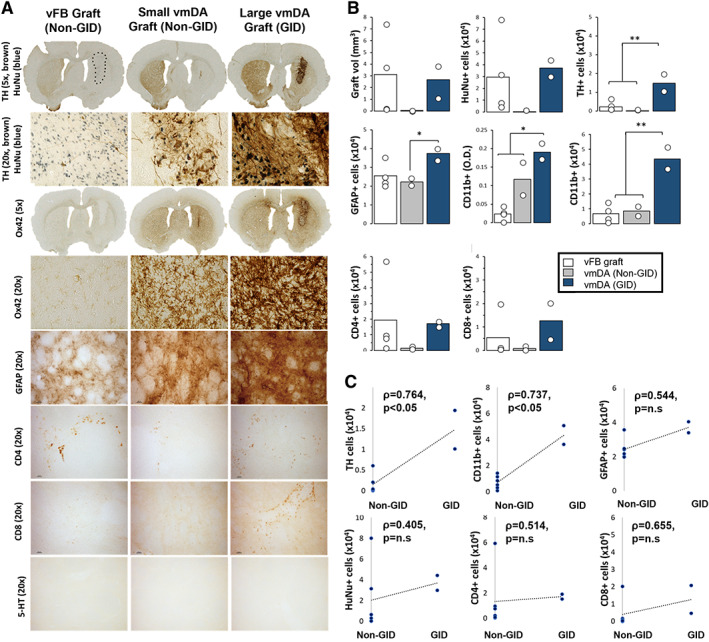
(**A**) Immunohistochemical analysis of hESC‐derived vmDA and vFB grafts. Dopamine neurons (tyrosine hydroxylase [TH], Millipore, MAB318, 1:2000; brown DAB) and human nuclei (HuNu, Millipore, MAB1281, 1:1000; blue VectorSG) at 5× and 20× magnification in vFB grafts, small vmDA grafts from non‐GID rats and large vmDA grafts from GID rats. The HuNu^+^ graft core of the vFB graft is delineated with a black dashed line. Activated microglia within the graft core and border (Ox42 [CD11b], Serotec, MCA275G, 1:1000) at 5× and 20× magnification in each graft group. Immunolabeling of reactive astrocytes (GFAP, DAKO, Z0334, 1:1000), CD4^+^ t‐lymphocytes (Abcam, Ab33775, 1:100), CD8^+^ t‐lymphocytes (Serotec, MC4A48G, 1:500) and 5‐HT^+^ neuron staining (Immunostar, 20,080, 1:10000). 5‐HT^+^ “control” staining in the raphe nucleus is shown in the left panel, whereas the center and right panels demonstrate no positive staining in the small or large vmDA grafts. (**B**) Quantification of cells in hESC‐derived vmDA and vFB grafts. Graft volume, total HuNu^+^ cells, TH^+^ cells within the graft, GFAP^+^ cells within the striatum, Ox42/CD11b optical density within the graft core/border, Ox42 cells/mm^3^ within the graft core/border, CD4^+^ cells around the perimeter of the graft and CD8^+^ cells around the perimeter of the graft were quantified for vFB grafts, small vmDA grafts (non‐GID rats) and large vmDA grafts (GID rats). GID rats had grafts with more TH^+^ cells than vFB or small vmDA grafts [F_2,5_ = 10.45, *P* < 0.05; GID vs. non‐GID and vFB, ps = 0.01] and more GFAP^+^ cells than grafts in non‐GID rats [F_2,5_ = 4.20, *P* < 0.05; GID vs. non‐GID, *P* < 0.05]. More activated microglia (Ox42^+^/CD11b^+^) cells were evident in GID rats than in non‐GID or vFB grafted rats [F_2,5_ = 21.50, *P* < 0.05; GID vs. non‐GID and vFB, ps < 0.05]. No other group effects were significant. (**C**) Correlations between grafted cells and GID behaviors. Significant correlations were revealed between GID and TH^+^ and Ox42^+^ cells. No correlations were revealed between GID the total HuNu cell count, GFAP, CD4^+^ or CD8^+^ cells. For 5× magnification images, all scale bars = 100 μm; For 20× magnification images, all scale bars = 1000 μm. vFB = ventral forebrain control grafts; vmDA = ventral midbrain ventral mesencephalic grafts. Error bars = standard errors of the mean. *P ≤ 0.05, **P ≤ 0.01, ***P ≤ 0.001.

Larger vmDA grafts were identified in GID‐expressing rats and small vmDA grafts in non‐GID rats. The vFB grafts were a similar volume to large vmDA grafts, containing similar numbers of human cells, but with an absence of mature DA neurons (Fig. 2A,B). Similar numbers of CD4^+^ and CD8^+^ t‐lymphocytes were present in large vFB and vmDA grafts, but more reactive astrocytes were observed in GID vmDA rats (Fig. 2A,B). No 5‐HT^+^ cells were observed in any of the grafts (Fig. 2A), despite positive control staining evident within the raphe nuclei.

Large vmDA grafts were associated with high levels of microglial activation in both hemispheres, whereas small vmDA grafts induced modest microglial activation in both hemispheres. In contrast, large vFB grafts induced almost no microglial activation (Fig. 2A,B). Neither graft volume, total HuNu^+^ cells, GFAP, CD4^+^, nor CD8^+^ t‐lymphocyte infiltration correlated with the development of spontaneous AIMs (Fig. 2C). High DA neuron content (tyrosine hydroxylase [TH]) and markedly increased microglial activation (Ox42) correlated significantly with GIDs (Fig. 2C).

## Discussion

This is the first report of measurable spontaneous GIDs occurring following transplantation of hESC‐derived vmDA neurons into an animal model. These behaviors occurred without prior exposure to l‐dopa or LID “priming” and with grafts devoid of 5‐HT neurons. Given that they were abolished by the D_2_ receptor antagonists eticlopride and by buspirone, which acts as a D_2_ receptor antagonist at high doses, this suggests that DA itself, and particularly D_2_‐like family receptors, play a role in mediating GIDs.

These long‐term (1 year post‐graft) pilot data are important insofar as they suggest that GID emergence is possible in clinical trials of hESC‐derived vmDA grafts. This is despite the evidence that hESC‐derived cell preparations for clinical use will contain no/limited 5‐HT neurons and patient selection will prioritize patients without severe LIDs. Important parallels can be drawn with recently published details on five patients who developed persistent GIDs in a double‐blind United States (US) fetal cell transplant study.^5^ Both of these datasets challenge current assumptions in the field, which suggest that GIDs expression may be dependent on 5‐HT expression.^24^, ^25^, ^26^, ^27^ Dopamine is directly implicated in both studies: (1) the presentation of GIDs resembles some elements of classic LIDs; (2) anti‐dopaminergic drugs reduce GIDs; (3) GIDs correlate with measures of dopamine (fluorodopa in clinical trials/TH^+^ immunohistochemistry here), and (4) GIDs occur in association with functional improvements (Unified Parkinson Disease Rating Scale [UPDRS] in clinical studies, amphetamine rotations here). Although in the present study, grafts contained ~14,000 TH^+^ cells (whereas rats have ~10–12,000 TH^+^ cells/hemisphere),^28^, ^29^ in patients GIDs have been induced from grafts of ~37,000 TH cells^5^ (human brain harbors ~170,000 substantia nigra pars compacta [SNpc] DA neurons)^30^ and without evidence of excess dopamine in PET scans.

There has remained an unanswered question about the temporal pattern of GID emergence clinically. One supposition is that it relates to the withdrawal of immunosuppression, potentially triggering inflammatory drivers,^6^ which would be consistent with data demonstrating that DA can mediate immune responses.^31^ In the current study, the large microglial inflammatory response was specific to brains with vmDA‐containing grafts although a direct causal relationship between neuroinflammation and GIDs could not be determined. Alternatively, GID emergence could relate to the time that it takes for DAergic neurons to begin significant maturation and outgrowth as a similar timescale occurred in a trial, which did not immunosuppress graft recipients.^5^

The use of neonatal desensitization makes this the first behavioral study of extended (12 months) human vmDA graft survival times in immunologically intact animals (in the absence of immunosuppression). Graft survival was low in this study (n = 4 grafts/group), but variability within this model has been reported previously.^32^, ^33^ Moreover, all published literature using this model has reported graft survival for a maximum of 6 months post‐graft.^20^, ^22^ The data presented here are, to our knowledge, the first report of human‐to‐rat graft survival up to 12 months post‐graft. Even if protection conferred by neonatal desensitization may reduce with time, this model has nevertheless been highly effective in revealing a previously unobserved phenomenon not readily investigated in standard immune‐compromised/suppressed animals.

The severity of spontaneous GIDs in these rats were mild‐to‐moderate in magnitude when compared to LID established in standard protocols in rodents^21^ and did not appear to worsen over the observed time. Importantly, if LIDs are not the sole driver for GIDs, then the risks to late‐stage patients may not be significantly elevated, whereas their benefits, such as reductions in LID, could be substantially more than early‐stage patients. Therefore, this therapy may be open to a broader population of people with PD.

Importantly for future clinical trials, although stem‐cell based therapies can be designed to be completely devoid of 5‐HT neurons and, therefore, may be considered safer from a GID perspective,^24^ our data suggest that this may not completely eliminate the risk for GIDs. The mechanism of action of spontaneous GIDs needs further investigation in larger studies, but if they can be mediated through excess dopamine and/or inflammatory responses, it may be possible to mitigate these through design of the therapeutic delivery.

## Conclusion

This is the first study to demonstrate that stem cell‐based therapies can induce spontaneous GIDs in a rodent model of PD, simultaneous with functional recovery. The data demonstrate that GID onset can occur independently of l‐dopa exposure and 5‐HT neurons in the graft. Instead, the data presented here suggest involvement of D_2_‐like family receptors and suggest that GIDs may be associated with graft inflammation.

## Author Roles

(1) Research project: A. Conception, B. Organization, C. Execution; (2) Statistical Analysis: A. Design, B. Execution, C. Review and Critique; (3) Manuscript: A. Writing of the First Draft, B. Review and Critique

E.L.L.: Research project: Conception, Organization, Execution; Manuscript: Writing of the First Draft, Review and Critique.

D.J. H.: Research project: Conception, Organization, Execution; Manuscript: Review and Critique.

E.R.V.: Research project: Execution; Statistical Analysis: Execution; Manuscript: Review and Critique.

R.L.H.: Research project: Execution; Manuscript: Writing of the First Draft and Review and Critique.

S.T.: Research project: Organization, Execution; Manuscript: Review and Critique.

M.J.L.: Research project: Conception, Organization, Execution; Statistical Analysis: Design, Execution, Review and Critique; Manuscript: Writing of the First Draft, Review and Critique.

## Financial Disclosures

MJL was supported by a Parkinson's UK Senior Research Fellowship (F1502); ELL was support by MRC grant MR/R00630X/1.

## Supporting information


**Video S1** Two rats are shown in this video, neither of whom have received any drug treatment at the point of videoing. These are spontaneous behaviors. Both rats have 6‐OHDA lesions in the right medial forebrain bundle. Grafted non‐GID rat: behaviors displayed by this rat typify those of 6‐OHDA lesioned rats, with ipsilateral tendencies (locomoting in a clockwise direction) with limited deviation of the head to tail line from the neutral straight with exploratory behaviors of sniffing and occasional rearing. Grafted GID rat: behaviors displayed by this rat reveal persistent driven contralateral locomotion (counterclockwise) with slow axial twisting involving repeated deviation of the head to tail line to a 45°–90° angle. The mild GIDs also consisted of repeated fast tapping of the contralateral forelimb, head bobbing, and orolingual movements, including tongue protrusions.

## Data Availability

Data available on request from the authors

## References

[1] Barker RA , Barrett J , Mason SL , Björklund A . Fetal dopaminergic transplantation trials and the future of neural grafting in Parkinson's disease. Lancet Neurol 2013;12:84–91. 23237903 10.1016/S1474-4422(12)70295-8

[2] Li W , Englund E , Widner H , et al. Extensive graft‐derived dopaminergic innervation is maintained 24 years after transplantation in the degenerating parkinsonian brain. Proc Natl Acad Sci 2016;113:6544–6549. 27140603 10.1073/pnas.1605245113 PMC4988567

[3] Hagell P , Piccini P , Björklund A , et al. Dyskinesias following neural transplantation in Parkinson's disease. Nat Neurosci 2002;5:627–628. 12042822 10.1038/nn863

[4] Olanow CW , Gracies J‐M , Goetz CG , et al. Clinical pattern and risk factors for dyskinesias following fetal nigral transplantation in Parkinson's disease: a double blind video‐based analysis. Mov Disord 2009;24:336–343. 19006186 10.1002/mds.22208

[5] Greene PE , Fahn S , Eidelberg D , Bjugstad KB , Breeze RE , Freed CR . Persistent dyskinesias in patients with fetal tissue transplantation for Parkinson disease. npj Parkinson's Dis 2021;7:38 33893319 10.1038/s41531-021-00183-w PMC8065148

[6] Piccini P , Pavese N , Hagell P , et al. Factors affecting the clinical outcome after neural transplantation in Parkinson's disease. Brain 2005;128:2977–2986. 16246865 10.1093/brain/awh649

[7] Soderstrom KE , Meredith G , Freeman TB , et al. The synaptic impact of the host immune response in a parkinsonian allograft rat model: influence on graft‐derived aberrant behaviors. Neurobiol Dis 2008;32:229–242. 18672063 10.1016/j.nbd.2008.06.018 PMC2886670

[8] Soderstrom KE , O'Malley JA , Levine ND , Sortwell CE , Collier TJ , Steece‐Collier K . Impact of dendritic spine preservation in medium spiny neurons on dopamine graft efficacy and the expression of dyskinesias in parkinsonian rats. Eur J Neurosci 2010;31:478–490. 20105237 10.1111/j.1460-9568.2010.07077.x PMC2940228

[9] Politis M , Wu K , Loane C , et al. Serotonergic neurons mediate dyskinesia side effects in Parkinson's patients with neural transplants. Sci Transl Med 2010;2:38ra46 10.1126/scitranslmed.3000976 20592420

[10] Pagano G , Niccolini F , Politis M . The serotonergic system in Parkinson's patients with dyskinesia: evidence from imaging studies. J Neural Transm 2018;125:1217–1223. 29264660 10.1007/s00702-017-1823-7 PMC6060863

[11] Shin E , Garcia J , Winkler C , Björklund A , Carta M . Serotonergic and dopaminergic mechanisms in graft‐induced dyskinesia in a rat model of Parkinson's disease. Neurobiol Dis 2012;47:393–406. 22579773 10.1016/j.nbd.2012.03.038

[12] Politis M , Oertel WH , Wu K , et al. Graft‐induced dyskinesias in Parkinson's disease: high striatal serotonin/dopamine transporter ratio. Mov Disord 2011;26:1997–2003. 21611977 10.1002/mds.23743

[13] Aldrin‐Kirk P , Heuer A , Wang G , Mattsson B , Lundblad M , Parmar M , Björklund T . DREADD modulation of transplanted DA neurons reveals a novel parkinsonian dyskinesia mechanism mediated by the serotonin 5‐HT6 receptor. Neuron 2016;90:955–968. 27161524 10.1016/j.neuron.2016.04.017 PMC4893163

[14] Lane EL , Vercammen L , Cenci MA , Brundin P . Priming for L‐DOPA‐induced abnormal involuntary movements increases the severity of amphetamine‐induced dyskinesia in grafted rats. Exp Neurol 2009;219:355–358. 19393238 10.1016/j.expneurol.2009.04.010

[15] Steece‐Collier K , Collier TJ , Danielson PD , Kurlan R , Yurek DM , Sladek JR Jr . Embryonic mesencephalic grafts increase levodopa‐induced forelimb hyperkinesia in Parkinsonian rats. Mov Disord 2003;18:1442–1454. 14673880 10.1002/mds.10588

[16] Kriks S , Shim J‐W , Piao J , et al. Dopamine neurons derived from human ES cells efficiently engraft in animal models of Parkinson's disease. Nature 2011;480:547–551. 22056989 10.1038/nature10648 PMC3245796

[17] Kirkeby A , Grealish S , Wolf DA , et al. Generation of regionally specified neural progenitors and functional neurons from human embryonic stem cells under defined conditions. Cell Rep 2012;1:703–714. 22813745 10.1016/j.celrep.2012.04.009

[18] Lelos MJ , Morgan RJ , Kelly CM , Torres EM , Rosser AE , Dunnett SB . Amelioration of non‐motor dysfunctions after transplantation of human dopamine neurons in a model of Parkinson's disease. Exp Neurol 2016;278:54–61. 26851542 10.1016/j.expneurol.2016.02.003 PMC4801014

[19] Grealish S , Heuer A , Cardoso T , et al. Monosynaptic tracing using modified rabies virus reveals early and extensive circuit integration of human embryonic stem cell‐derived neurons. Stem Cell Rep 2015;4:975–983. 10.1016/j.stemcr.2015.04.011 PMC4471831 26004633

[20] Heuer A , Kirkeby A , Pfisterer U , Jönsson ME , Parmar M . hESC‐derived neural progenitors prevent xenograft rejection through neonatal desensitisation. Exp Neurol 2016;282:78–85. 27235932 10.1016/j.expneurol.2016.05.027 PMC4920671

[21] Breger LS , Dunnett SB , Lane EL . Comparison of rating scales used to evaluate l‐DOPA‐induced dyskinesia in the 6‐OHDA lesioned rat. Neurobiol Dis 2013;50:142–150. 23072976 10.1016/j.nbd.2012.10.013

[22] Kelly CM , Precious SV , Scherf C , et al. Neonatal desensitization allows long‐term survival of neural xenotransplants without immunosuppression. Nat Methods 2009;6:271–273. 19270699 10.1038/nmeth.1308

[23] Loane C , Politis M . Buspirone: what is it all about? Brain Res 2012;1461:111–118. 22608068 10.1016/j.brainres.2012.04.032

[24] Henchcliffe C , Parmar M . Repairing the brain: cell replacement using stem cell‐based technologies. J Parkinsons Dis 2018;8:S131–S137. 30584166 10.3233/JPD-181488 PMC6311366

[25] Barker RA , TRANSEURO Consortium . Designing stem‐cell‐based dopamine cell replacement trials for Parkinson's disease. Nat Med 2019;25:1045–1053. 31263283 10.1038/s41591-019-0507-2

[26] Doi D , Samata B , Katsukawa M , et al. Isolation of human induced pluripotent stem cell‐derived dopaminergic progenitors by cell sorting for successful transplantation. Stem Cell Rep 2014;2:337–350. 10.1016/j.stemcr.2014.01.013 PMC3964289 24672756

[27] Fan Y , Winanto , Ng SY . Replacing what's lost: a new era of stem cell therapy for Parkinson's disease. Transl Neurodegener 2020;9:2 31911835 10.1186/s40035-019-0180-x PMC6945567

[28] Nair‐Roberts RG , Chatelain‐Badie SD , Benson E , White‐Cooper H , Bolam JP , Ungless MA . Stereological estimates of dopaminergic, GABAergic and glutamatergic neurons in the ventral tegmental area, substantia nigra and retrorubral field in the rat. Neuroscience 2008;152:1024–1031. 18355970 10.1016/j.neuroscience.2008.01.046 PMC2575227

[29] Healy‐Stoffel M , Omar Ahmad S , Stanford JA , Levant B . Differential effects of intrastriatal 6‐hydroxydopamine on cell number and morphology in midbrain dopaminergic subregions of the rat. Brain Res 2014;1574:113–119. 24924804 10.1016/j.brainres.2014.05.045 PMC4115364

[30] Chu Y , Kompoliti K , Cochran EJ , Mufson EJ , Kordower JH . Age‐related decreases in Nurr1 immunoreactivity in the human substantia nigra. J Comp Neurol 2002;450:203–214. 12209851 10.1002/cne.10261

[31] Matt SM , Gaskill PJ . Where is dopamine and how do immune cells see it?: dopamine‐mediated immune cell function in health and disease. J Neuroimmune Pharmacol 2019;15:114–164. 10.1007/s11481-019-09851-4 31077015 PMC6842680

[32] Janowski M , Jablonska A , Kozlowska H , et al. Neonatal desensitization does not universally prevent xenograft rejection. Nat Methods 2012;9:856–858. 22936164 10.1038/nmeth.2146 PMC3432986

[33] Roberton VH , Rosser AE , Kelly CM . Neonatal desensitization for the study of regenerative medicine. Regen Med 2015;10:265–274. 25933236 10.2217/rme.14.76

